# Lower levels of the neuroprotective tryptophan metabolite, kynurenic acid, in users of estrogen contraceptives

**DOI:** 10.1038/s41598-023-43196-6

**Published:** 2023-09-29

**Authors:** Anne-Lise Bjørke-Monsen, Kristin Varsi, Sunniva Todnem Sakkestad, Arve Ulvik, Cathrine Ebbing, Per Magne Ueland

**Affiliations:** 1 Laboratory of Medical Biochemistry, Innlandet Hospital Trust, Lillehammer, Norway; 2 Laboratory of Medical Biochemistry, Førde Central Hospital, Førde, Norway; 3 Department of Medical Biochemistry and Pharmacology, Haukeland University Hospital, Bergen, Norway; 4 Department of Infection, Haukeland University Hospital, Bergen, Norway; 5 Bevital AS, Bergen, Norway; 6 Department of Obstetrics and Gynecology, Haukeland University Hospital, Bergen, Norway

**Keywords:** Biochemistry, Biomarkers, Medical research

## Abstract

Changes in kynurenine metabolites are reported in users of estrogen containing contraception. We have assessed kynurenines, vitamin B6, vitamin B2 and the inflammation markers, C-reactive protein (CRP) and neopterin, in healthy, never-pregnant women between 18 and 40 years (*n* = *123*) and related this to their use of hormonal contraception. The population included 58 women, who did not use hormonal contraceptives (non-users), 51 users of estrogen-containing contraceptives (EC-users), and 14 users of progestin only contraceptives (PC-users). EC-users had significantly lower plasma kynurenic acid (KA) and higher xanthurenic acid (XA) levels compared to non-users. Serum CRP was significantly higher and negatively associated with both vitamin B6 and B2 status in EC-user compared to non-users. No significant differences in any parameters were seen between PC-users and non-users (*p* > 0.1). The low KA and high XA concentration in users of estrogen containing contraception resemble the biochemical profile observed in vitamin B6 deficiency. The hormonal effect may result from interference with the coenzyme function of vitamin B6 and B2 for particular enzymes in the kynurenine metabolism. KA has been suggested to be neuroprotective and the significantly reduced concentration in EC-users may be of importance in the observed increased risk of mood disorders among users of oral contraceptives.

## Introduction

Hormone contraceptive use is linked to changes in basal neuroendocrine and inflammatory profiles, potentially increasing the sensitivity to mood disturbances ^1^. Oral contraceptives contain either a combination of estrogen and progesterone or progesterone alone. Both hormones have been related to mood alterations^2^ and epidemiologic data have suggested an increased risk for incident depression among users of oral contraceptives, particularly in adolescents ^3,4^.

Estrogens are also reported to affect tryptophan (Trp) degradation through the kynurenine (Kyn) pathway^5,6^, and changes in Kyn metabolites have been implicated in the pathogenesis of neuropsychiatric disorders ^7–9^.

**Figure 1:**
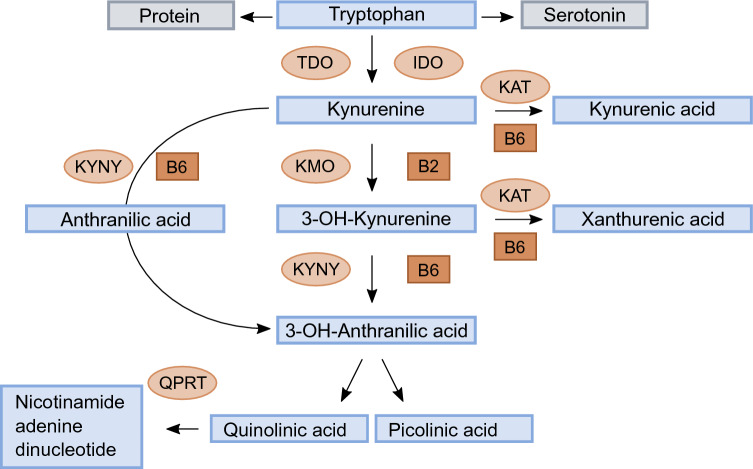
Schematic drawing of the tryptophan metabolism through the kynurenine pathway. Enzymes: tryptophan 2,3-dioxygenase (TDO), indole 2,3-dioxygenase (IDO), kynurenine aminotransferase (KAT), kynurenine 3-monooxygenase (KMO) kynureninase (KYNU), quinolinate phosphoribosyl transferase (QPRT). Cofactors: vitamin B6 (pyridoxal 5´-phosphate, PLP) and vitamin B2 (flavin adenine dinucleotide, FAD).

The essential amino acid Trp is mainly (90%) metabolised to Kyn, while a small portion serves as precursor of serotonin (Fig. 1). Conversion of Trp to Kyn is catabolized by tryptophan 2,3-dioxygenase (TDO) or indole 2,3-dioxygenase (IDO). The activity of TDO is activated mainly by cortisol, while IDO is stimulated by proinflammatory cytokines, like interferon gamma, tumor necrosis factor alpha (TNF-a) and interleukin-6. Kyn is further metabolised by the enzyme kynurenine aminotransferase (KAT) to kynurenic acid (KA) or by kynurenine 3-monooxygenase (KMO) to 3-hydroxykynurenine (HK)^10^. HK can be converted to xanthurenic acid (XA) by KAT, or metabolised through 3- hydroxyanthranilic acid (HAA) by kynureninase (KYNU) to either picolinic acid (Pic) or quinolinic acid (QA). Kyn can also be converted to anthranilic acid (AA) by the enzyme KYNU.

The enzymes KAT and KYNU require vitamin B6 in the form of pyridoxal 5′-phosphate (PLP) as a cofactor, while KMO requires vitamin B2 in the form of flavin adenine dinucleotide (FAD) as a cofactor. Deficiency of either vitamins will cause disturbances in the kynurenine metabolites ^11^. In patients with low levels of PLP, the concentrations of KA will be low, while HK, XA and QA will be high compared to healthy controls. Deficiency of PLP will therefore increase the ratio between HK and KA, termed HK/KA ratio, as well as the ratio between HK and the sum of kynurenic acid (KA) + anthranilic acid (AA) + xanthurenic acid (XA) + 3-hydroxyanthranilic acid (HAA), termed HK ratio (HKr), a proposed marker of vitamin B6 deficiency ^12^.

Treatment with estrogen-containing oral contraceptives are reported to cause elevated urinary excretions of XA, Kyn, HK, and HAA, changes which are similar to the changes observed in dietary vitamin-B6 deficiency ^13^. The use of combined oral contraceptives has also been reported to increase circulating C-reactive protein (CRP) concentrations^14^. Plasma PLP is the most commonly used marker of vitamin B6 status and has consistently been shown to be low in inflammatory conditions^15^.

Although oral contraceptives have been used for more than 50 years by millions of women, there are still questions regarding the biological consequences of these medications^16^. In order to study estrogen-related changes in Kyn metabolism, we have investigated Trp, Kyn metabolites, inflammation markers and B vitamins in healthy, never-pregnant women aged 18 to 40 years in relation to their use of hormonal contraception.

## Results

### Demographics

The total study population included 158 healthy, never-pregnant women between 18 and 40 years. In order to diminish variation in Trp intake, women with a special diet were excluded (n = 35) and the 123 women, who were included in the study, were all omnivores.

Demographic data according to reported current use of contraceptives (non-users, n = 58, PC-users, n = 14 and EC-users, n = 51) are given in Table 1. A minority were regular users of micronutrient supplements and users of tobacco. Apart from a higher consumption of alcohol in women who used hormonal contraceptives compared to non-users, there were no significant differences in demographic data between the three groups (Table 1, Supplemental Table 1).

**Table 1:** Baseline characteristics of healthy women according to use of hormonal contraceptives (n = 123).

(25th-75th percentile)	Non-users N = 58	Users of hormonal contraceptives		*P* value
		Progestin-only N = 14	Estrogen and progestin N = 51	
Age, years, median (IQR)	24 (22–29)	24 (22–25)	24 (22–26)	0.71^1^
Body mass index, kg/m^2^, median (25th-75th percentile)	22.2 (20.8–24.7)	20.8 (20.0–21.6)	22.3 (20.5–23.5)	0.06^1^
Regular users of micronutrient supplements, n (%)	12 (21%)	1 (7%)	14 (28%)	0.26^2^
Smokers^3^, n (%)	1 (2%)	1 (7%)	7 (14%)	0.06^2^
Alcohol, number of units/week, median (25th-75th percentile)	1.0 (0.4–3.0)	4.0 (1.0–4.3)	2.0 (1.0–4.0)	0.01^1^

^1^Comparison by Kruskal–Wallis test.

^2^Comparison by Pearson Chi-square test.

^3^Smokers defined as plasma cotinine ≥ 85 nmol/L.

### Inflammation markers and vitamins according to use of hormonal contraceptives

There were significant differences in the concentrations of serum CRP and plasma neopterin according to use of hormonal contraception (Table 2; Supplemental Table 2). EC-users had the highest median serum CRP concentration and the lowest median plasma neopterin concentrations compared to PC-users and non-users. Serum CRP in EC-users was significantly higher (*p* = 0.007) compared to non-users and PC-users (*p* = 0.002), while plasma neopterin was lower, compared to non-users (*p* = 0.04) and PC-users (*p* = 0.009) (Supplemental Table 2). No significant differences were seen in CRP or neopterin concentrations between non-users and PC-users (*p* ≥ 0.07) (Supplemental Table 2).

**Table 2:** Concentrations of inflammation parameters, vitamins and tryptophan/kynurenine metabolites according to use of hormonal contraceptives (n = 123).

Parameters Median (25th-75th percentile)	Non-users N = 58	Users of hormonal contraceptives		*P* value^1^
		Progestin-only N = 14	Estrogen and progestin N = 51	
Markers of inflammation, serum/plasma				
C-reactive protein, mg/L	1.0 (0.6–2.0)	1.0 (0.7–1.0)	2.0 (1.0–5.0)	< 0.001
Neopterin, nmol/L	11.9 (9.6–14.5)	14.7 (10.9–19.0)	10.1 (8.4–13.8)	0.01
Vitamins, plasma				
Pyridoxal 5-phosphate, nmol/L	63.8 (51.2–94.3)	91.9 (58.0–108.0)	58.1 (42.7–103.0)	0.07
Pyridoxal, nmol/L	11.6 (9.0–15.0)	14.5 (10.6–19.5)	11.4 (8.4–15.6)	0.13
Pyridoxic acid, nmol/L	20.5 (19.8–26.6)	25.1 (15.6–39.9)	22.3 (15.3–28.1)	0.69
PAr2	27 (22–34)	25 (18–31)	32 (24–40)	0.04
Riboflavin, nmol/L	8.9 (5.9–14.1)	9.2 (4.6–12.1)	7.4 (5.7–13.0)	0.65
Flavin mononucleotide, nmol/L	11.4 (8.8–15.5)	15.6 (12.0–18.7)	12.0 (9.1–17.6)	0.04
Nicotinamide, nmol/L	191 (146–264)	188 (138–235)	202 (147–271)	0.72
Tryptophan and kynurenine metabolites, plasma				
Tryptophan, µmol/L	70.7 (59.6–81.1)	72.7 (65.3–84.3)	70.4 (62.9–82.7)	0.62
Kynurenine, µmol/L	1.48 (1.33–1.64)	1.56 (1.37–1.77)	1.43 (1.30–1.62)	0.30
3-Hydroxykynurenine, nmol/L	41.7 (35.5–50.8)	42.7 (35.2–49.1)	43.2 (35.4–54.7)	0.92
Kynurenic acid, nmol/L	52.8 (41.5–65.8)	60.9 (47.0–73.3)	38.1 (28.7–50.4)	< 0.001
Anthranilic acid, nmol/L	13.1 (11.0–16.2)	15.3 (10.1–18.0)	12.3 (10.0–15.3)	0.15
3-Hydroxyanthranilic acid, nmol/L	45.4 (39.0–57.1)	52.3 (39.7–63.9)	50.3 (36.6–64.3)	0.64
Xanthurenic acid, nmol/L	18.0 (13.3–22.5)	18.1 (12.8–25.0)	23.7 (15.5–29.5)	0.07
Picolinic acid, nmol/L	55.3 (49.0–71.7)	63.5 (53.5–85.7)	64.7 (41.3–73.7)	0.50
Quinolinic acid, nmol/L	336 (268–383)	321 (287–383)	342 (299–425)	0.35
Kynurenine metabolite ratios				
Kynurenine/Tryptophan ratio^3^	21 (19–25)	21 (18–24)	20 (18–22)	0.12
Kynurenic acid/Quinolinic acid^3^	17 (13–22)	17 (14–21)	11 (9–13)	< 0.001
HKr^4^	33 (28–38)	29 (27–33)	36 (29–43)	0.01

^1^Comparison by Kruskal–Wallis test.

^2^PAr: Pyridoxic acid/( Pyridoxal 5-phosphate + Pyridoxal). The ratio was multiplied by 100.

^3^The ratio was multiplied by 100.

^4^ HKr: 3-Hydroxykynurenine/(Kynurenic acid + Anthranilic acid + 3-Hydroxyanthranilic acid + Xanthurenic acid). The ratio was multiplied by 100.

There were no significant differences in B6 and B2 vitamers between EC-user and non-users (*p* > 0.2), but the PAr index was higher in EC-users (*p* = 0.03) (Table 2, Supplemental Table 2). The highest median plasma PLP concentration was seen in PC-users and the lowest in EC-users, while the opposite was seen for the PAr index. PC-users had higher median FMN concentration compared to non-users (*p* = 0.01), but not to EC-users (*p* = 0.07) (Table 2, Supplemental Table 2).

### Correlations between inflammatory marker and B-vitamers

Serum CRP was significantly negative correlated to plasma PLP, PL PA, riboflavin and FMN in EC-users (rho: − 0.3, − 0.5, *p* < 0.01) and non-users (rho = − 0.3–− 0.4, *p* < 0.03). No significant correlations were seen between CRP and B6 and B2 species in PC-users.

Plasma neopterin was significantly positive correlated to the PAr index in EC-users (rho: 0.4, *p* = 0.005), but no other significant correlations were seen between neopterin and B6 and B2 species in any group.

### Kynurenine metabolites according to use of hormonal contraceptives

Plasma concentrations of Trp and Kyn metabolites according to use of hormonal contraception are given in Table 2. The lowest median plasma KA concentrations and highest median plasma XA concentrations were seen in EC-users. Median plasma KA concentration was 28% lower, while median plasma XA concentration was 32% higher in EC-users compared to non-users and the median ratio between KA and QA was reduced by 35% (Table 2, Supplemental Table 2). There were no significant differences in median plasma Trp, Kyn and any other Kyn metabolite between EC-users and non-users (*p* > 0.08) (Table 2).

Compared to PC-users, EC-users had lower median plasma KA concentrations and KA/QA ratio (Table 2, Supplemental Table 2).

There were no significant differences in any Kyn-related parameters between PC-users and non-users (*p* > 0.1) (Table 2).

### Vitamin B6 status by PLP and HKr

Plasma PLP was significantly negative correlated to HKr in EC-users (rho: − 0.6, − 0.5, *p* < 0.001), PC-users (rho: − 0.7, − 0.5, *p* < 0.001), and non-users (rho = − 0.3, *p* < 0.009). Median HKr was increased by 9% (*p* = 0.04) in EC-users compared to non-users, indicative of impaired vitamin B6 status related to use of estrogen containing contraception.

### Correlations involving KA and XA

Plasma KA and XA were not significantly related to serum CRP or plasma neopterin in any groups. Plasma KA was positive correlated to PLP (rho: 0.3, *p* = 0.07), riboflavin and FMN (rho = 0.3, *p* = 0.02) in EC-users, to PLP (rho = 0.3, *p* = 0.3), riboflavin (rho: 0.7, *p* = 0.008), FMN (rho = 0.5, *p* = 0.09) in PC-users, and weakly only to PLP (rho = 0.2, *p* = 0.08) in non-users.

No significant correlations were observed of plasma XA with B6 and B2 vitamers in EC-users (rho < 0.2, *p* =  > 0.2), weaker correlations were seen for PLP( rho: 0.3, *p* = 0.3) and riboflavin (rho: 0.5, *p* = 0.06) in PC-users, and more significant correlations to PLP (rho = 0.4, *p* = 0.004), riboflavin (rho: 0.2, *p* = 0.1) and FMN (rho = 0.3, *p* = 0.04) were seen in non-users.

### Predictors of KA and XA

Plasma Kyn was the strongest predictor for plasma KA (beta = 0.37, *p* < 0.001), followed by use of hormonal contraceptives (beta = − 0.29, *p* = 0.001) and serum CRP (beta = -0.19, *p* = 0.04), in a multiple linear regression model which additionally included age, BMI, use of alcohol and smoking (plasma cotinine), plasma Trp, PLP and FMN. Including plasma neopterin in the model did not change the results. By applying stepwise regression, only use of hormonal contraceptives (beta = -0.39 *p* < 0.001), plasma Kyn (beta = 0.35, *p* < 0.001) and PLP (beta = 0.21, *p* = 0.008) remained as predictors for plasma KA.

The strongest predictor for XA in the full multiple linear regression model was plasma cotinine (beta = − 0.26, *p* = 0.009), plasma Trp (beta = 0.25, *p* = 0.02), followed by use of hormonal contraceptives (beta = 0.23, *p* = 0.02). Including plasma neopterin did not change the models. By applying stepwise regression, plasma Trp (beta = 0.34, *p* < 0.001), plasma cotinine (beta = − 0.23, *p* = 0.01) and use of hormonal contraceptives (beta = 0.20, *p* = 0.03) remained as significant predictors for plasma XA.

## Discussion

In healthy, never-pregnant women aged 18 to 40 years, use of estrogen containing contraception was associated with significantly lower plasma concentrations of KA and higher concentrations of XA compared to non-users of hormonal contraception.

Compared to non-users, in EC-users serum CRP was higher and all vitamin B6 and B2 species were significantly negatively associated with CRP and positive related to plasma KA.

Estrogen has a number of effects on the modulation of the inflammatory response and immune cell function ^17^, and many of these are mediated by estrogen receptors present on monocytes and macrophages^18,19^. CRP is mainly produced in hepatocytes in response to increased levels of inflammatory cytokines, especially interleukin-6^20^, while neopterin is synthesized by monocytes and macrophages in response to interferon-γ produced by activated T cells ^21^. Median concentrations of CRP and neoterin differed according to use of contraception. EC-users had higher serum CRP, and lower plasma neopterin compared to non-users, which is in accordance with published data ^14,22,23^. The use of combined oral contraceptives and postmenopausal hormone therapy have repeatedly been reported to elevate circulating CRP concentrations^14,17,24^. A reduced concentration of neopterin by estrogen therapy in menopause has been reported by several authors^18,22,23^, but data on neopterin concentrations in premenopausal women using hormonal therapy is not available.

In inflammatory conditions, plasma PLP and liver PLP are low, while erythrocyte and muscle PLP are unaffected and functional vitamin B6 biomarkers are not changed ^15^. Transfer of vitamin B6 to the sites of inflammation, where it may serve as a co-factor in pathways producing metabolites with immunomodulating effects, has therefore been suggested as a cause of low plasma PLP observed in inflammation ^15^. Also increased catabolism of the vitamin in inflammatory conditions have been proposed as a possible explanation. An increase in PA, the vitamin B6 catabolite that is excreted into the urine, relative to PL plus PLP measured as the PAr index has been interpreted as evidence of increased vitamin B-6 catabolism^11^. More than 90% of the variations in the PAr index are reported to be explained by inflammatory markers such as CRP, neopterin, white blood cell count and the Kyn to Trp ratio, indicating that there might be an increased catabolism of vitamin B-6 during inflammation^25^. We observed a significant negative correlation between CRP and PLP, while there was a positive correlation between CRP and the PAr index in EC-users, data which might support the hypothesis of increased B6 catabolism in inflammatory conditions.

Significant negative correlations were also observed between CRP and riboflavin and FMN in both EC-users and non-users. Riboflavin plays multiple roles in human health, and has recently been proposed as a possible new antimicrobial agent, as the vitamin may suppress or inactivate the growth of different microbes including bacteria, viruses, fungi and parasites ^26^. Data on change in riboflavin and FMN concentrations in inflammatory conditions are, however, to our knowledge unavailable.

The EC-users in our study had a 9% lower median plasma PLP and 9% higher HKr, indicating a poorer vitamin B6 status in EC-users. A recent study found a 10% lower median plasma PLP level in oral contraceptive users compared to non-users^27^, an observation in agreement with our results. Oral contraceptives with estrogens have been reported to reduce the level of several vitamins, including B6 and B2 ^28–31^, however most of these studies were published more than 40 years ago when the level of estrogen in oral contraceptives were much higher^32^. The lower levels of B6 vitamin in oral contraceptive users have also been suggested to be due to tissue redistribution of PLP, rather than actual vitamin B-6 deficiency^27,28,30^. However, our observation of an increase in the functional B6 marker HKr in EC users suggests an impaired B6 status in these women.

We observed significantly lower plasma KA and higher plasma XA concentrations in women taking estrogen containing hormonal contraceptives compared to non-users. Meier et al. found significantly lower levels of KA in women taking oral contraceptives compared to non-users and the same has been reported by others ^27,33^. Increased urinal excretion of XA has also been reported in women taking estrogen containing contraceptives^34^.

The observed profile in EC-users is similar to the biochemical profile observed in vitamin B6 deficiency, with low KA and high XA and HKr^35^. One prevailing hypothesis for the metabolic changes resembling B6 deficiency in EC-users is that estrogen conjugates, which are typically elevated during pregnancy, inhibit the binding of PLP to both KAT and KYNU^36,37^. A marked fall in the excretion of XA after pyridoxine treatment has been observed in women taking progestogen-estrogen, suggesting that the hormonal effect may result from interference with the coenzyme function of PLP for these particular enzymes^38^.

There are neuroactive metabolites in the kynurenic pathway, including the *N*-methyl-d-aspartate (NMDA) receptor antagonist KA and agonist QA^15^. KA is the only naturally occurring antagonist of the glutamatergic NMDA receptor in the human brain^39^ and has under experimental conditions been shown to be neuroprotective^40^. A main finding in our study is lower KA concentrations and KA/QA ratio in EC-users compared to non-users. A recent meta-analysis reported that kynurenic acid and the KA/QA ratio were decreased in mood disorders, such as major depressive disorder, bipolar disorder and schizoaffective disorder^41,42^.

By only including women with an omnivore diet, we reduced the possible impact of low B6 vitamin and Trp intake, which might have affected kynurenine metabolism. As we did not have information about the menstrual phase or plasma sex hormone concentrations we were unable to relate our data to plasma estrogen concentrations, which is a limitations of this study. Additionally, the low number of PC-users made it difficult to test the progestin effect on kynurenine metabolism.

In healthy, never-pregnant women aged 18 to 40 years, use of estrogen containing contraception was associated with significantly lower plasma concentrations of KA and higher concentrations of XA, compared to non-users of hormonal contraception. The reason for this is unknown, however, the hormonal effect may result from interference with the coenzyme function of vitamin B6 and B2 for particular enzymes in the kynurenine metabolism.

KA has been suggested to be a neuroprotective amino acid ^40^ and the significantly reduced KA concentration in EC-users may be related to the reported increased risk for mood disorders among users of oral contraceptives.

## Material and methods

### Study population and design

Between June 2012 and March 2015 never-pregnant women aged 18 to 40 years were asked to participate in a study on factors related to B-vitamin status in healthy women. Recruitment was done among employees and students at Haukeland University Hospital and the University of Bergen, Norway and no specific randomization method was applied.

Ethical approval of the protocol was granted by the Regional Committee for Medical Research Ethics Western Norway (2011/2447). All procedures were performed in accordance with the relevant guidelines and regulations. Written informed consent was obtained from all women.

### Clinical data

The women completed a questionnaire concerning age, body weight, height, health status, diet, as well as use of multiple micronutrient supplements, alcohol, tobacco and use of hormone containing contraceptives,. Women who had a chronic disease and used daily medications were excluded.

Regular use of micronutrient supplements was defined as use more than three days per week and the definition of a regular tobacco user was based on a plasma cotinine concentration > 85 nmol/L ^43^.

### Use of hormone containing contraceptives

Use of hormonal contraception, including oral contraceptives, hormone implants and injections, was recorded. Progestins-only contraceptives contain different forms and doses of progestins, while combination contraceptives additionally contain ethinylestradiol in the range 20–35 µg ^44^.

Women who did not use hormonal contraception were defined as non-users, women who used estrogen containing contraceptives were defined as the EC-users, whereas women who used progestins-only contraceptives were defined as the PC-users.

### Blood sampling and analysis

Non-fasting blood samples were obtained by antecubital venipuncture and collected into Vacutainer Tubes with (for plasma) and without (for serum) EDTA (Becton Dickinson). EDTA tubes were placed in ice water, and plasma was separated within 4 h. The samples were stored at − 80 °C until analysis.

Analysis Trp, Kyn metabolites, vitamin B2 (riboflavin and flavin mononucleotide (FMN)) and B6 vitamers (PLP, pyridoxal and pyridoxic acid), cotinine and neopterin was performed by liquid chromatography on an Agilent series 1100 HPLC system coupled with electrospray ionization tandem mass spectrometry (ESI–MS/MS) on an API 4000 triple-quadrupole tandem mass spectrometer from Applied Biosystems/MSD SCIEX^45^ at Bevital, Bergen, Norway (www.bevital.no).

Pic was added to an existing method based on LC–MS/MS ^45^, using multiple reaction monitoring (MRM) in the positive mode, recoding the ion pairs 124 m/z–78 m/z for picolinic acid and 128 m/z–88 m/z for picolinic-d4 acid (internal standard). The within-day and between CV were 6–7% and 5–8%, respectively (Supplemental file 1).

QA was added to an existing method based on LC–MS/MS ^45^, using multiple reaction monitoring (MRM) in the positive mode, recoding the ion pairs 168 m/z 78 m/z for quinolinic acid and 171 m/z–81 m/z for quinolinic-d3 acid (internal standard). The within-day and between CV were 7.0–7.5% and 7.2–10.3%, respectively (Supplemental file 2).

Serum CRP concentrations were determined using an immunoturbidimetric method using a Cobas 8000 analyzer (Roche diagnostics, Milan, Italy).

### Statistical analysis

Results are presented as median and interquartile range (IQR), and compared by Kruskal Wallis test with Dunn’s test for multiple pairwise comparisons. For variables and metabolites that reported a p value of < 0.05, post hoc Dunn’s tests were performed to investigate differences between the individual study groups. Chi-square test was used for categorical data. Spearman correlations and multiple linear regression models were used to explore relationships between parameters. For KA and XA, we interrogated a panel of predictors known to modify serum concentrations of kynurenines, including age, BMI, use of hormonal contraception, alcohol and tobacco (plasma cotinine), serum CRP, plasma Trp, Kyn, PLP and FMN ^11,33,46–48^ in a multiple linear regression model for the whole study population. Further inclusion of neopterin did not change the estimates. Stepwise regression was applied as an exploratory analysis.

The SPSS statistical program (version 29) was used for statistical analyses*.* Two-sided *p* values < 0.05 were considered statistically significant.

### Supplementary Information

Supplementary Tables.

Supplementary Information.

## Data Availability

The datasets used and/or analysed during the current study are available from the corresponding author on reasonable request.
