# Supplementary material for: Lower levels of the neuroprotective tryptophan metabolite, kynurenic acid, in users of estrogen contraceptives
Source: Sci Rep. 2023 Sep 29;13:16370. doi: 10.1038/s41598-023-43196-6 (PMC10541439; doi:10.1038/s41598-023-43196-6)
Supplement: Supplementary file 1 — Supplementary Tables. [file 41598_2023_43196_MOESM1_ESM.docx]

| **Supplemental Table 1. Summary of Kruskal-Wallis univariate analysis** | | | | |
| --- | --- | --- | --- | --- |
| Parameters | Kruskal-Wallis test  p value | Dunn’s post hoc test p value | | |
|  |  | EC-users - Non-users | EC-users - PC-users | Non-users – PC-users |
| Age, years | 0.71 | * | * | * |
| Body mass index, kg/m^2^ | 0.06 | * | * | * |
| Alcohol, number of units/week | 0.01 | 0.02 | 0.34 | 0.01 |

*Metabolites with a p value of > 0.05 did not undergo a post hoc Dunn’s test

| **Supplemental Table 2. Summary of Kruskal-Wallis univariate analysis** | | | | |
| --- | --- | --- | --- | --- |
| Parameters | Kruskal-Wallis test  p value | Dunn’s post hoc test p value | | |
|  |  | EC-users - Non-users | EC-users - PC-users | Non-users – PC-users |
| **Markers of inflammation** | | | | |
| C-reactive protein | <0.001 | 0.007 | 0.002 | 0.11 |
| Neopterin | 0.01 | 0.04 | 0.009 | 0.07 |
| **Vitamins** | | | | |
| Pyridoxal 5-phosphate | 0.07 | * | * | * |
| Pyridoxal | 0.13 | * | * | * |
| Pyridoxic acid | 0.69 | * | * | * |
| PAr^2^ | 0.04 | 0.03 | 0.08 | 0.55 |
| Riboflavin | 0.65 | * | * | * |
| Flavin mononucleotide | 0.04 | 0.29 | 0.07 | 0.01 |
| Nicotinamide | 0.72 | * | * | * |
| **Tryptophan and kynurenine metabolites** | | | | |
| Tryptophan | 0.62 | * | * | * |
| Kynurenine | 0.30 | * | * | * |
| 3-Hydroxykynurenine | 0.92 | * | * | * |
| Kynurenic acid | <0.001 | <0.001 | 0.002 | 0.55 |
| Anthranilic acid | 0.15 | * | * | * |
| 3-Hydroxyanthranilic acid | 0.64 | * | * | * |
| Xanthurenic acid | 0.07 | * | * | * |
| Picolinic acid | 0.50 | * | * | * |
| Quinolinic acid | 0.35 | * | * | * |
| **Kynurenine metabolite ratios** | | | | |
| Kynurenine/Tryptophan ratio^3^ | 0.12 | * | * | * |
| Kynurenic acid/Quinolinic acid ^3^ | <0.001 | <0.001 | 0.01 | 1.00 |
| HKr^4^ | 0.01 | 0.07 | 0.02 | 0.07 |

*Metabolites with a p value of > 0.05 did not undergo a post hoc Dunn’s test
