# Supplementary material for: Lower levels of the neuroprotective tryptophan metabolite, kynurenic acid, in users of estrogen contraceptives
Source: Sci Rep. 2023 Sep 29;13:16370. doi: 10.1038/s41598-023-43196-6 (PMC10541439; doi:10.1038/s41598-023-43196-6)
Supplement: Supplementary file 2 — Supplementary Information. [file 41598_2023_43196_MOESM2_ESM.pdf]

BEVITAL
Assessment of disease risk
by targeted metabolomics

Supplementary data on method for analysis of Picolinic acid (Pic)

Method based on article
Midttun et al (2009), PMID 19337982.

Material
Picolinic acid (purity >98%) was obtained from Sigma-Aldrich, St.Louis, MO 63178 USA.
2 Picolinic-d4 acid (purity >98%) was obtained from CDN isotopes. Ponte-Claire, Quebec, Canada.

Instrumentation
Same as in PMID 19337982.

Chromatography and detection
LC-MS/MS; positive-ion multiple reaction monitoring (MRM); retention time = 2.25 min.
Picolinic acid precursor ion = 124 m/z; product ion = 78 m/z.
Picolinic-d4 acid precursor ion = 128 m/z; product ion = 82 m/z.

Method performance
Linear range: 8 - 400 nmol/L.
Linearity: r2: 0.99.
LOD (S/N >5): 8 nmol/L.
Within-day CV: 6-7 %.
Between-day CV: 5-8 %.

The B-vitamin network with ramification to the tryptophan-niacin pathway

Midttun Ø[1,*], Kvalheim, G[1,2] and Ueland PM[2,3]
[1]Bevital A/S, Bergen Norway.
[2]Section for Pharmacology, Institute of Medicine, University of Bergen, Norway.
[3]Laboratory of Clinical Biochemistry, Haukeland University Hospital, Bergen, Norway
[*]Corresponding author, email nkjbm@uib.no

Background: Vitamins B2, B6, folate and B12 function as coenzymes in the one-carbon metabolism, and vitamins B2 and B6 also in the tryptophan-niacin (vitamin B3) pathway (Figure 1). Metabolism of tryptophan through this pathway is increased in inflammation. One form of niacin, nicotinamide, is converted to N^1-methylnicotinamide by using S-adenosylmethionine as methyl donor, thereby connecting one-carbon metabolism with the tryptophan-niacin pathway. In addition, published data on plasma concentrations of vitamin B3 forms (non-supplemented), quinolinic acid and N^1-methylnicotinamide are sparse.

Aim: To include additional tryptophan-niacin pathway metabolites in an established HPLC-MS/MS assay that measures vitamins B2 and B6, several tryptophan metabolites, and inflammation markers. Further, to use a panel of plasma biomarkers to investigate relations between B-vitamin status, one-carbon metabolism, the tryptophan-niacin pathway, and inflammation.

Results: We included the tryptophan-niacin pathway metabolites quinolinic acid, nicotinamide, nicotinic acid and N^1-methylnicotinamide in an established HPLC-MS/MS assay (1). 60 µL of plasma was deproteinized and the supernatant injected onto a stable-bond C8 column. The chromatographic run-time was 5 minutes (Figure 2). The detector was a triple quadropole mass spectrometer fitted with electrospray probe and operated in positive multiple reaction monitoring mode. A total of 21 plasma/serum analytes (Table 1) are measured by the assay, and assay capacity is 172 samples/day. Method validation characteristics (Table 2) make this assay suitable for use in routine analysis of endogenous plasma concentrations (Table 3).

Conclusions: The expanded assay will be used to analyse plasma samples from biobanks. Combined with other assays, this will allow investigation of the metabolic network involving of B-vitamins, one-carbon metabolism, the tryptophan-niacin pathway and inflammation.

Acknowledgements: Marit Krokeide is thanked for excellent technical assistance in assay development.

References:

(1) Midttun et al., Rapid Communications in Mass Spectrometry vol. 23, 2009, p. 1371.

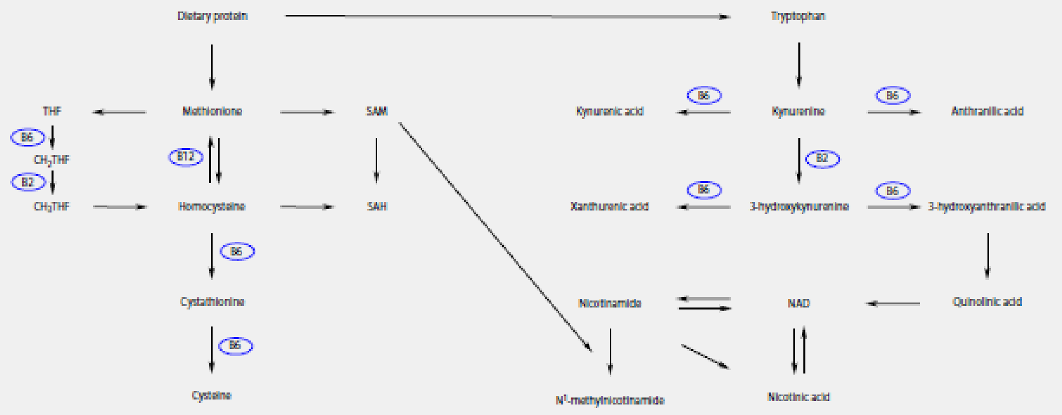

Figure 1. One-carbon metabolism (left) and the tryptophan - niacin pathway (right).
SAM function as methyl donor for the convertion of nicotinamide to N^1-methylnicotinamide. Coenzymes are shown in blue ellipses. Abbreviations: THF, tetrahydrofolate; CH_2THF, methylenetetrahydrofolate; CH_3THF, methyltetrahydrofolate; SAM, S-adenosylmethionine, SAH, S-adenosylhomocysteine, NAD, nicotinamide adenine dinucleotide; B2, vitamin B2; B6, vitamin B6; B12, vitamin B12.

Table 1. Analytes included in the assay.

Vitamin B2 forms:	Riboflavin	Tryptophan metabolism:	Tryptophan
	Flavin mononucleotide		Kynurenine
Vitamin B6 forms:	Pyridoxal 5'-phosphate		Kynurenic acid
	Pyridoxal		Anthranilic acid
	4-pyridoxic acid		3-hydroxykynurenine
	Pyridoxine		Xanthurenic acid
	Pyridoxamine		3-hydroxyanthranilic acid
Other:	Cystathionine		Quinolinic acid
	Cotinine		Nicotinamide
	Neopterin		Nicotinic acid
			N^1-methylnicotinamide

Table 2. Method validation characteristics of the new analytes in the assay.

Analyte	Within-day CV (%)[a]	Between-day CV (%)[a]	Recovery (%)[b]	Linear range (nM)	Linearity (r^2)	LOD (nM)
Quinolinic acid	7.0 - 7.5	7.2 - 10.3	88 - 96	1.6 - 4000	0.993	1.6
Nicotinamide	10.0 - 12.2	8.7 - 11.8	105 - 109	20 - 16000	0.987	20
Nicotinic acid	9.9 - 17.3	10.7 - 14.8	106 - 112	20 - 8000	0.995	20
N^1-methylnicotinamide	4.8 - 6.6	6.8 - 8.5	92 - 98	5 - 16000	0.997	5.0

[a]The CV experiments (N=18) were performed at three concentrations (low, medium and high) for each analyte. Low was an unspiked plasma pool having concentrations of 269.6 nM quinolinic acid, 116.8 nM nicotinamide, 67.9 nM nicotinic acid and 67.0 nM N^1-methylnicotinamide, medium was spiked with 1000 nM quinolinic acid, nicotinamide and N^1-methylnicotinamide and 100 nM nicotinic acid, while high was spiked with 2000 nM quinolinic acid, nicotinamide and N^1-methylnicotinamide and 200 nM nicotinic acid.
[b]Recovery was calculated from the CV experiments.

Table 3. Plasma concentrations (nM)[a].

Analyte	Median	(5,95 percentile)
Quinolinic acid	225.5	(133.6, 383.8)
Nicotinamide	177.4	(91.9, 388.3)
Nicotinic acid	64.3	(42.9, 101.3)
N^1-methylnicotinamide	68.5	(27.1, 220.5)

[a]Measured in 172 presumed healthy adult humans.

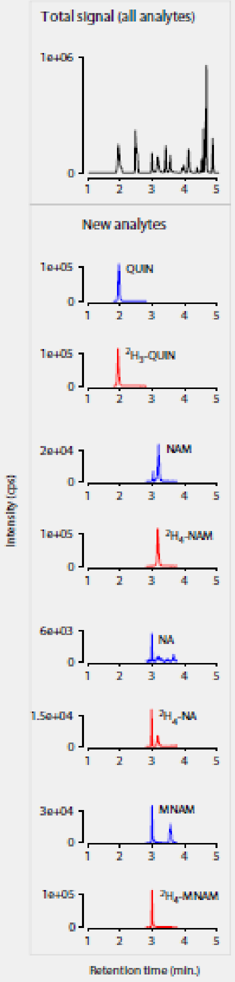

Figure 2. Chromatogram.
Chromatogram of unspiked plasma pool (Table 2). Abbreviations: cps, counts per second; QUIN, quinolinic acid; NAM, nicotinamide; NA, nicotinic acid; MNAM, N^1-methylnicotinamide.
